# Substance P and Chronic Pain in Patients with Chronic Inflammation of Connective Tissue

**DOI:** 10.1371/journal.pone.0139206

**Published:** 2015-10-07

**Authors:** Barbara Lisowska, Aleksander Lisowski, Katarzyna Siewruk

**Affiliations:** 1 Department of Anaesthesiology, Medical Centre for Postgraduate Education, Adam Gruca Clinical Hospital, Postgraduate Medical Education Centre, Otwock, Poland; 2 Faculty of Production Engineering, Warsaw University of Life Sciences, Warsaw, Poland; 3 Faculty of Veterinary Medicine, Warsaw University of Life Sciences, Warsaw, Poland; Queen Mary University of London, UNITED KINGDOM

## Abstract

**Objective:**

Evidence suggests that substance P (SP) is involved in chronic joint inflammation, such as the pathogenesis of rheumatoid arthritis and osteoarthritis. The goal of the research was to evaluate the correlation between chronic pain and changes in the SP level in patients with chronic inflammation of the connective tissue.

**Methods:**

Patients with osteoarthritis and rheumatoid arthritis were enrolled in this study. The relationship between chronic pain intensity and the serum SP concentration was evaluated in these groups of patients with osteoarthritis and rheumatoid arthritis.

**Results:**

The results showed a positive correlation between the serum SP concentrations and chronic pain intensity.

**Conclusions:**

1. The SP serum concentration was significantly different between the groups of patients with OA and RA. 2. There was a positive correlation between the serum SP concentration and chronic pain intensity in OA and RA patients.

## Introduction

Von Euler and Gaddun discovered substance P (SP) in 1931 [1]. SP is a well-known neuropeptide that is widely distributed in both the central (CNS) and periphery (PNS) nervous systems.

The induction and transmission of nociceptive signals causes the release of SP from sensory fibres in the spinal cord [2].

Substance P is also released from many cells of the immune system, such as macrophages, lymphocytes and dendritic cells. There are data on the activity of SP and its involvement in the inflammatory response [3], [4], together with other peripheral cells, including bone cells [5].

Rheumatoid arthritis (RA) is a systemic autoimmune disease associated with chronic inflammation of the connective tissue.

Substantial recent evidence suggests that SP and its receptors are involved in joint inflammation and are associated with some aspects of the pathophysiology of RA [6], [7]. Chronic pain is associated with arthritis and the involvement of substance P through the NK–1 receptor. Studies on NK–1 have shown a positive correlation between the size and severity of joint destructive changes and the pain and density of NK–1 receptors in inflamed tissues [8], [9].

Considering this aim, we evaluated the intensity of pain and the serum SP concentrations in patients with chronic inflammatory connective tissues. Chronic inflammation is a common symptom in osteoarthritis (OA) and RA.

## Patients and Methods

Patients with OA or RA were randomly enrolled in the study.

Osteoarthritis was diagnosed according to the symptoms and results of X-ray joint examination.

Patients with RA were fully diagnosed according to the criteria of the American Rheumatism Association [10].

All patients were regularly taking non-steroidal anti-inflammatory drugs (NSAIDs).

The exclusion criteria were as follows:

- Weight: BMI > 30
- Severe liver or kidney disease
- The presence of other inflammatory diseases

For all patients with RA, the disease activity was assessed using both subjective and objective methods. The total painful and swollen joint numbers, according to the 28-joint index were assessed using subjective grading. The sedimentation according to the Westergren procedure (mm/h) was assessed as the objective grade. The DAS 28 (Disease Activity Score) according to four variables was estimated using the DAS 28 formula with DAS 28 Calculator v1.1-beta (Alfons & Michiel).

The intensity of pain was evaluated using a numeric pain scale (NPS) from 0 to 5, where 0 corresponded to no pain, 1 –slight, 2 –moderate, 3 –intensive 4 –severe and 5 –worst imaginable pain. The patients performed the evaluation.

The concentration of SP was measured in all collected serum samples. The 4–5 ml venous blood samples were collected into the serum test tubes, delivered to an analytical laboratory and centrifuged (2000 rpm/10 min). The sera were placed into 200 μl test tubes and stored at -70°C. The SP concentration was measured using the Substance P Immunoassay Test (R&D System) in accordance with the instructions from the manufacturer. The minimum detectable level for this assay was 8.0 pg/ml.

The immunological and laboratory tests were conducted at the Department of Immunopathology and the Diagnostic Laboratory at the Institute of Rheumatology in Warsaw.

### Statistics

STATISTICA v.10 was used to perform all statistical analysis. Because the NPS values are quality variables, nonparametric tests were used. Differences between the variables for the factors were analysed using the Mann-Whitney *U* test. The correlation analyses were performed using the Spearman rank correlation test. To identify interdependencies, further tests were performed to find a regression function, the power of which was checked by the Snedecor F test. *P* values < 0.05 were considered significant.

Factor analysis was performed to find patterns among the measured variables. Factor loadings > 0.70 were considered. The factor analyses were performed with Varimax, using Kaiser normalisation as a rotation method.

The Medical Ethics Committee of Institute of Rheumatology in Warsaw approved the protocol. The experiments were conducted according to the principles expressed in the Declaration of Helsinki.

Written informed consent was obtained from all patients.

## Results

Seventy patients suffering from chronic pain due to OA or RA were classified.

These patients were divided into two groups according to their primary disease (OA–Osteoarthritis or RA–Rheumatoid Arthritis). In OA group were 23 patients and RA group included 47 patients. OA patients had a mean ±SD age 63±12 years while RA patients had a mean ±SD age 55±11 years. In RA patients the disease lasted a mean ±SD 17±7.18 years and the disease activity according to DAS28 was a mean ±SD 5.2±1.16.

In both groups, there were more women than men. There was a significant difference (*P* < 0.005) in the patients’ mean age between the groups.

### Correlation between SP and NPS in the OA and RA groups

The preoperative cardiopulmonary status of all patients was almost the same. All patients were ASAII according to the physical status classification system of the American Society of Anaesthesiologists.

The mean SP serum concentration, standard deviation, limit values and pain severity in both groups are shown in Table 1. A statistically significant difference between the OA and RA groups was only found for SP (*P* = 0.016, Table 1).

**Table 1:** Serum concentration of substance P and severity of chronic pain in both groups of patients.

Parameter/group	OA			RA			*P-*value
	Mean ±SD	Min	Max	Mean ±SD	Min	Max	
SP, pg/ml	327±290	46	1000	563±425	50	1500	0.016
NPS, points	3.0±0.8	2	4	3.1±0.8	2	4	0.582

There was a correlation between SP and NPS (r_s_ = 0.531; *P* < 0.05), but it was stronger for the OA group (r_s_ = 0.725; *P* < 0.05) than for the RA group (r_s_ = 0.423; *P* < 0.05).

### Relationship between NPS and SP in the OA group

**Fig 1:**
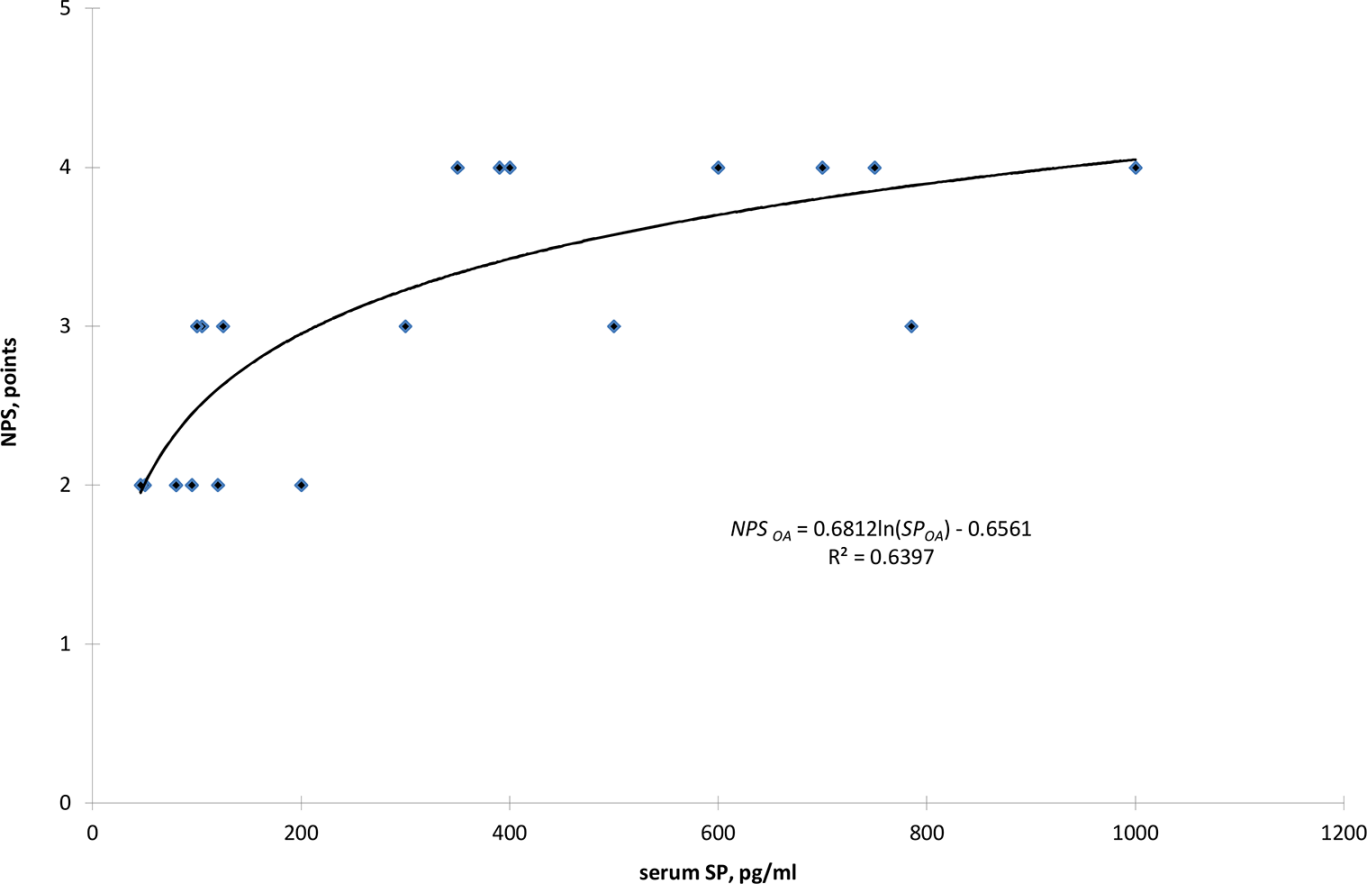
The relationship between NPS and the serum concentration of substance P (SP) for OA patients.

Based on a correlation analysis and positional relationship between the SP and NPS points, a logarithmic function was found only for the OA group patients (Fig 1). The value of the determination coefficient indicates that 63.97% of the SP concentration alters the perceived chronic pain according to NPS. In the range of low concentrations of P, up to 200 pg/ml, there was a dynamic increase in the value of perceived pain NPS, in the range of 2 to 3 points.

For an SP concentration of 200 pg/ml, a turning point can be observed with a change in the dynamics of an appreciable increase in pain, which is expressed on a point scale. With respect to the changes in the SP concentration from 200 pg/ml to 1200 pg/ml, the pain felt by patients changed by only one point, increasing in value from 3 to 4 points.

### Correlation between SP, NPS, DAS, age and disease duration in the RA group

A positive correlation between DAS and NPS (0.612), SP and NPS (0.426), SP and DAS (0.365), and age and RA disease years for the group of RA patients is shown in Table 2. Based on the positive correlation between the independent variables of DAS and SP, the two variables contain similar information, but the value of the correlation coefficient between DAS and NPS (0.612) is higher than that between SP and NPS (0.429), indicating that the DAS disease activity has a larger positive influence on the severity of chronic pain compared with the SP concentration.

**Table 2:** The results of analysis of correlation on the Spearman rank test between SP, chronic pain in NPS, DAS, age and time of disease in years.

Parameter	Age	RA years	DAS	NPS	SP
Age	1.000				
RA years	0.302^a^	1.000			
DAS	0.017	0.000	1.000		
NPS	-0.125	-0.127	0.612^a^	1.000	
SP	-0.155	0.067	0.365^a^	0.426^a^	1.000

^a^–correlation with *P* <0.05.

Factor analysis performed on the RA group yielded two factors with eigenvalues > 1, explaining 66% of the total variation between the measured variables (Table 3). The DAS, NPS, and SP were grouped together with the larger weighting on DAS and NPS. There was also an interrelationship between age and disease duration in years.

**Table 3:** Factor analyses on SP, NPS, DAS, age and RA years’ disease.

Variable	Factor	
	1	2
Age		0.784
RA years		0.787
DAS	0.843	
NPS	0.849	
SP	0.744	

For logarithmic values of SP and all values of DAS, the regression analysis showed that ln (SP) and DAS have a statistically significant effect on the severity of chronic pain, with a critical significance level of *P* = 0.049. In this full analysis, it appeared that the constant does not have a statistically significant effect (*P* = 0.808); therefore, it was removed from a regression function. The final results of the regression analysis without a constant are presented in Table 4. The results showed a high statistical impact of DAS and the logarithmic values of SP ln (SP) on NPS for *P* < 0.001 and *P* = 0.002.

**Table 4:** Results of the regression analysis for chronic pain intensity according to NPS depending on the disease activity score (DAS) and the logarithmic concentration of substance P–ln (SP) for the group of patients with RA after removing the intercept.

Parameter	*β*	Standard error of *β*	*b*	Standard error of *b*	t(43)	*P*-value
DAS	0.604	0.117	0.364	0.070	5.151	<0.001
Ln(SP)	0.387	0.117	0.208	0.063	3.298	0.002

Regression summary for the dependent variable NPS: correlation coefficient R = 0.9847, determination coefficient R^2^ = 0.9696, adjusted correlation coefficient R^2^ = 0.9682, values of the Fisher-Snedecor test F(2, 44) = 701.97, critical level *P*-value *P* < 0.001, standard error of estimate: 0.5776.

According to the given values of the regression coefficient, the following regression function was formed.

$$NPS_{RA}=0.36DAS+0.21\ln(SP)$$

**Fig 2:**
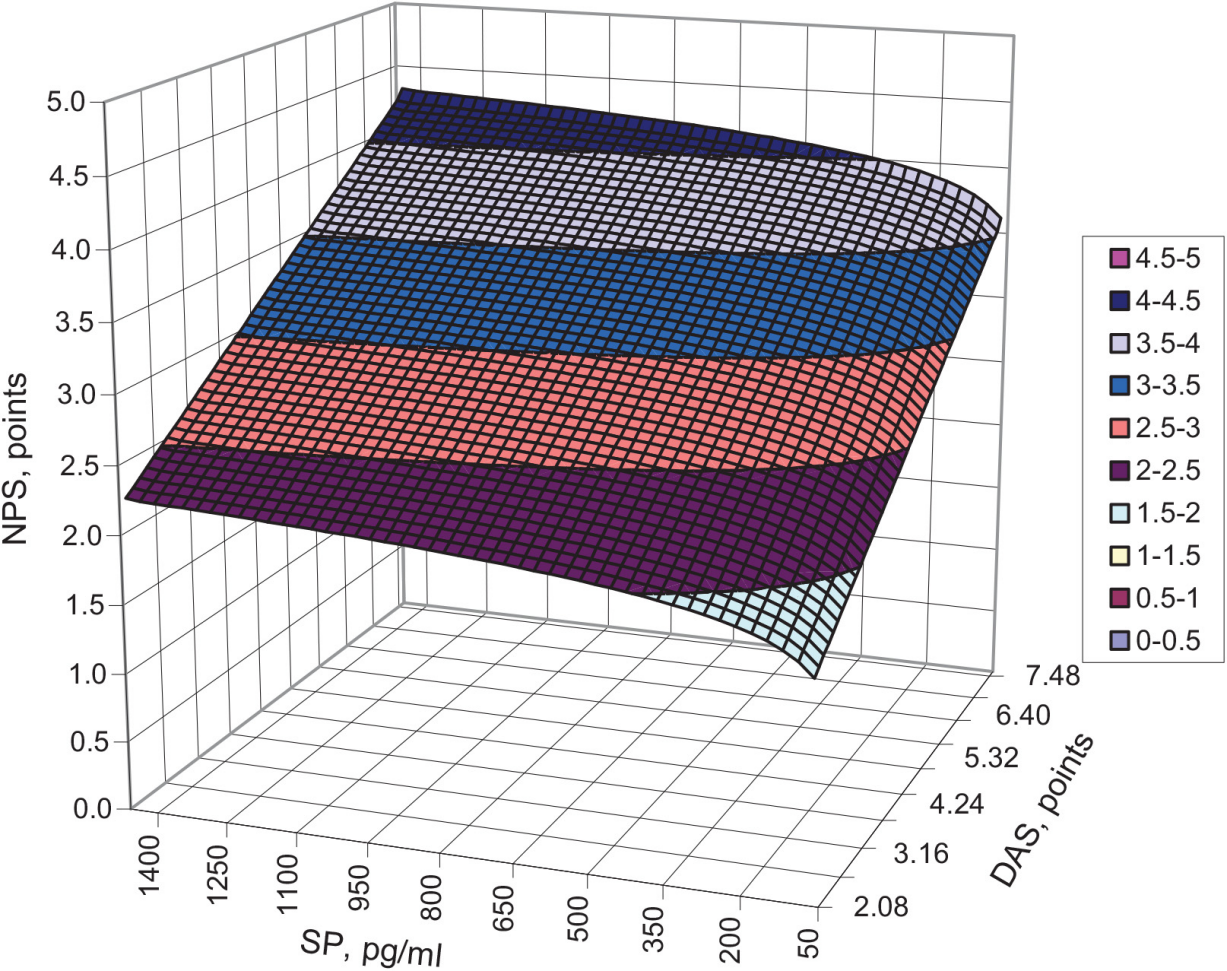
Interaction between the serum concentration of substance P (SP) and the disease activity (DAS) and intensity of pain (NPS) for patients with RA.

The value of an improved determination coefficient indicates that in 96.82% of this group of RA patients, both DAS and ln (SP) exert an influence on chronic pain severity. A graphical interpretation of the function of this surface is shown in Fig 2. The resulting graph shows that the chronic pain intensity was higher with an increasing concentration of substance P and increasing disease activity. Similar observations were made in the group of patients with OA with respect to an increasing SP concentration but not disease activity. For example, patients with SP concentrations greater than 350 pg/ml described the intensity of perceptible chronic pain as lower by an average of 0.5 points on the NPS scale.

This trend is extended over the entire range of disease activity (DAS), which is clearly visible in Fig 2, where the curve changes its curvature along the edge of DAS. The dynamic of the changes in NPS was higher for DAS than it was for SP, as evidenced by the plane angles of the respective Cartesian axes. The patients with lower activity in RA complained of lower pain ≤ 2 points, irrespective of the SP concentration (up to 1600 pg/ml), whereas the patients with high disease activity experienced more severe pain, even at a low SP serum concentration. The critical area of the pain intensity NPS above 3 points (severe pain) referred to patients with an SP concentration ranging from 1400 to 1600 pg/ml and an approximate DAS level of 4. This critical area was also achieved for patients with lower SP concentrations of 200 pg/ml when the disease activity was greater than 4 points. Disease activity, which was described using the DAS scale, had a greater impact on the dynamics of chronic pain than did the SP concentration in patients with RA.

## Discussion

The results obtained have indicated a high serum concentration of SP in all of the patients enrolled in this study. This finding suggests a connection with both the chronic inflammatory state and chronic pain, which was defined as pain lasting more than three months.

From the outcome of the investigation one can conclude that in the patients with OA, a high serum SP level can be treated as evidence supporting the role of inflammation in the pathogenesis of osteoarthritis. In addition the significantly higher serum SP level was demonstrated in RA patients compared with OA patients. The most likely explanation of the results is the connection between the long-lasting advanced auto immunological disease and systemic chronic inflammation.

Similarly, Grimholm et al. showed a correlation between high SP serum levels and long-lasting RA [11] whereas researchers have found high SP serum levels in RA patients [12], [13]. Based on the current literature, O'Connor and Baerwald described the inflammatory diseases in which SP participates and the contribution of the neuroendocrine system in the development of disease activity in RA patients.

Lambert et al. observed the direct pro inflammatory effect of SP on rheumatoid fibroblast-like synoviocytes (FLS) via NK–1 [14]. It has been shown that FLS are located in the synovial intimal lining and play a key role in cartilage destruction by producing cytokines. Furthermore, the action of SP may cause vasodilatation by the induction vascular cell adhesion molecule–1 (VCAM–1). The indirect pro inflammatory effect of SP is related to the regulation of pro inflammatory cytokines together with prostaglandin PGE_2_ released from inflammatory cells [15].

At the same time high SP levels and high NK–1 receptor expression were observed in the synovial fluid obtained from RA patients [16], [17], [18]. According to Lotz and Partsch, the addition of substance P into the synovial cell culture caused the release of many various factors, including prostaglandin E and collagenosis [19], [20]. Levine et al. showed the contribution of SP to the severity of adjuvant-induced arthritis with strong pain [21], [22]. Moreover, Matucci-Cerini et al. found a high concentration of SP in the synovial fluid of RA patients [23]. Straub and Cutolo also presented the action of SP as a pro inflammatory agent that is responsible for stimulating the release of pro inflammatory cytokines (IL–1, IL–2, and TNF) from various cells [24]. All of these studies concerned the participation of SP in RA development.

It is worth emphasising that this study also focused on patients with OA because there are few studies about the contribution of SP in the pathogenesis of osteoarthritis. Our results seem to confirm the essential role of SP in the inflammatory component of OA.

Patients with obesity were excluded from our study. Several clinical studies demonstrated the role of SP in the development of obesity, which is defined as a type of chronic and systemic inflammatory disease [25], [26], [27].

In our patients, the mean severity of chronic pain was classified as intensive (3 points), and there was no statistically significant difference between the evaluated groups of patients.

The positive correlation between the serum SP level and chronic pain intensity was found in both groups, but this correlation was clearly stronger in OA patients.

The obtained results for the value of the determination coefficient indicate that the pain intensity was dependent on the serum SP levels in 64% of the patients with OA. The remaining 36% of the cases could be accounted for by factors such as the nerve dysfunction associated with a long-lasting inflammatory state. Therefore, the presented results confirm the participation of SP in modulating the pain in both groups of patients with inflammatory diseases.

Referring to the group of patients with RA, a high correlation between disease activity and pain intensity has been shown. Disease activity, as determined using the DAS28 scale, appeared to have a greater impact on the intensity of chronic pain than did the SP concentration in patients with RA. These findings show that smaller values of DAS28, even at very high SP concentrations, correspond to less pain, whereas higher values of DAS28 are related to stronger pain, even at lower SP concentrations. This relationship is confirmed by clinical examination because pain is the major complaint of RA patients with high disease activity. In their studies, Garip and Eser assessed the connection among fatigue, disease activity, pain and functional status in RA patients [28], [29]. They confirmed the strong association between fatigue and the severity of pain, disease activity (DAS28) and the functional status. However, they did not assess these items in relation to the SP level.

In this study, we present the first evidence of a link among the DAS28, pain and SP.

In healthy tissues, SP has the unique feature of being released from only C type fibres with strong stimuli. In contrast, in inflamed tissues, which have many more receptors on neurons due to the inflammatory processes, the nocicepive impulse may be spontaneously trigged without external stimulation [30]. Therefore, in this situation, the release of SP may be enhanced in the inflamed tissue. Furthermore, the synovium of RA patients is characterised by a low density of sympathetic nerve fibres with an increased SP density on sensory nerve fibres compared with OA patients [31].

Confirming the correlation between chronic/acute pain and the SP serum concentration in our study should be an aim of further research on the use of the NK–1 antagonist as a component of new drugs for pain therapy. The hypothesis concerning the analgesic properties of the SP antagonist has yet to be proven.

## Conclusions

1. The SP serum concentration was significantly different between the groups of patients with OA and RA.
2. There was a positive correlation between the serum SP concentration and chronic pain intensity in OA and RA patients.
